# The promise of gene editing: so close and yet so perilously far

**DOI:** 10.3389/fgeed.2022.974798

**Published:** 2022-07-15

**Authors:** David J. Segal

**Affiliations:** Genome Center and Department of Biochemistry and Molecular Medicine, University of California, Davis, Davis, CA, United States

**Keywords:** challenges, regulations, innovation, *in-vivo* delivery, editors, costs

## Introduction

On the one hand, it is striking how the promise of genome editing is advancing. Regulatory restrictions have largely eased on genetically engineered crops that carry genome modifications that are similar to spontaneous mutations or those produced by conventional chemical or radiation-based methods (Van Vu et al., 2022). Plants produced by site-directed nuclease type 1 methods (SDN1), for which substitutions and indels are produced only by the action of the nuclease, have been deregulated in many countries. An exception are those countries within the European Union, where, despite being the third largest producer of genetically engineered crops behind China and the USA, SDN1 crops remain subject to the stringent regulations for genetically modified organisms (GMOs). Such stringent regulations are considered to have a dampening effect on agriculture innovation in the EU, and are perhaps similar to the dampening effect of long regulatory delays on the genetic engineering of livestock animals (Van Eenennaam et al., 2021). Since the first report of genetic engineering in livestock animals in 1985, only a single food animal has been commercialized. This is in part due to the USA Food and Drug Administration and their EU counterparts classifying any intentional altered genomic DNA in animals as an investigational new animal drug (INAD) that is not generally recognized as safe. However, there is a growing realization that the current EU policy towards SDN1 crops needs to be updated (Dima et al., 2022), giving hope to the wider use of these directed editing methods that can dramatically accelerate the production of new varieties compared to traditional breeding techniques.

Interestingly, regulations have not hindered innovation in the application of genetic engineering to human health. In fact, this area has been a significant driver of technological advances. Recent publications and scientific meetings, such as the Keystone Symposium on Precision Genome Engineering and the American Society for Gene and Cell Therapy Annual Meeting, highlight the rapid advances in genome editing tools, driven in large part by a sense that new treatments for human disease enabled by these tools are just around the corner. Indeed, by some estimates there are over 100 products using genome editors now in clinical trial (CRISPR Medicine News), led by companies, such as CRISPR Therapeutics, Intellia Therapeutics, Sangamo Therapeutics, Editas Medicine, Precision Biosciences, Caribou Biosciences, Locus Biosciences, and many others. In the academic sector, Phase 1 of the NIH Somatic Cell Genome Editing Consortium (Saha et al., 2021), which had focused primarily on developing new editors and delivery methods, has led to a Phase 2 that is primarily focused on using these tools to develop treatments up to the stage of submitting an Investigational New Drug (IND) application to the US Food and Drug Administration (FDA).

## Delivery challenges

The challenges posed by the demands of eventual human treatments have set several trajectories of innovation. A significant challenge remains the efficient delivery of the editor to the nucleus of the target cell. *Ex-vivo* therapies, such as those for sickle cell disease (SCD) or the production of T-cells carrying a chimeric antigen receptor (CAR T-cells), two of the major indications in this area, can take advantage of a variety of techniques (*e.g.*, electroporation or transfection using lipid nanoparticles [LNP] (Kazemian et al., 2022)) to introduce the editor in various molecular forms (*e.g.*, plasmid DNA, messenger RNA, or ribonucleoprotein complexes [RNP]). The more transient molecular forms, mRNA or RNPs, are generally preferred because plasmid DNA can stimulate innate immune responses in cells, and the long-term expression of the editor can lead to increased off-target effects and immune responses to the editor if expressed in the body. However, the challenges are greater for *in-vivo* therapies. Currently, viral vectors, such as adeno-associated viruses (AAV), remain the most efficient method to deliver editors to cells in the body. However, the limited packaging capacity of AAV poses a formidable constraint. Cas9 from *Staphylococcus aureus* (*Sa*Cas9), which is smaller than the more widely used Cas9 from *Streptococcus* *pyogenes* (*Sp*Cas), can just barely fit in an AAV along with an expression cassette for the single-guide RNA (sgRNA). Products such as the EDIT-101 from EDITAS Medicine has used this approach for the treatment of the eye disease Leber Congenital Amaurosis 10, which is now in clinical trial (Maeder et al., 2019). However, larger payloads such tissue-specific promoters, base editors, prime editors or epigenetic editors, are unable to be packaged in a single AAV particle. The challenge of delivering large editor systems has driven several lines of innovation. The Cas9 protein can be split, allowing a two-vector system to deliver N-terminal and C-terminal parts for reassembly in the cell. Though popular in preclinical studies, this approach is generally not favored for clinical applications. A second approach has been the discovery of even smaller Cas9 and Cas12 proteins. This effort has identified dramatically smaller proteins such as CasMINI (Xu et al., 2021)and CasΦ (Pausch et al., 2020), as well spawned companies dedicated to finding new CRISPR systems in metagenomic data such as Metagenomi and Arbor Biotechnology. A third approach is to abandon AAV in favor of LNP, which have the strong advantages of 1) much larger packaging capacity, 2) far less immune response to the particle (Kenjo et al., 2021), and 3) enable the use of the preferred transient molecular forms of the editor such as mRNA and potentially RNP. NTLA-2001 from Intellia Therapeutics has used this approach for the treatment of the liver disease Transthyretin Amyloidosis, which is now in clinical trials (Gillmore et al., 2021). The major limitation of LNP is that, currently, they are only efficient for delivery to the liver. However, there are exciting efforts by both academic and industry labs to engineer enhanced transduction capabilities for LNP (Qiu et al., 2021) as well as AAV (Challis et al., 2022), again giving hope to the wider use of these methods to dramatically improve delivery capabilities in the near future.

## Editor challenges

Aside from delivery, another significant challenge is the action of the editor itself. Early concerns about “off-target” editing have largely subsided, perhaps due to a better selection of guide-RNAs, short duration of editor expression, and improved methods for finding off-target events (Giannoukos et al., 2018). However, there are growing concerns about “unexpected events” on-target, such as translocations, very large deletions, loss of entire chromosomal arms, integrations of the viral vector, and chromothripsis (Weisheit et al., 2020; Leibowitz et al., 2021). While these events generally occur in <5% of edited cells, it is essentially a certainty that such events will occur among the million or so cells that are edited in a therapeutic treatment. The consequences of these unexpected events remain unclear, and proponents would point out that no adverse outcomes such as cancer have been observed in any preclinical or clinical trial. However, the concerns are sufficient to fuel increased interest in alternative strategies that do not create double-strand breaks in DNA, such as base, prime, transposon, epigenetic and RNA editors. Beam Therapeutics, Prime Medicine, Tessera Therapeutics, Integra Therapeutics, SalioGen Therapeutics, and Chroma Medicine are just some of the companies emerging in this space, and are now able to advance towards clinical trials due to the improved delivery systems for large payloads described above. However, many challenges remain. Base and prime editors allow safe and highly efficient mutation of genes, as in Intellia’s knock-out of *TTR* for Transthyretin Amyloidosis mentioned above (Gillmore et al., 2021). However, the promise of base and prime editing to correct mutations in genes is currently less clear, since there are often 10–100 mutated alleles reported for each gene. A change in regulation may again be the answer, if using the same editor with just a different guide-RNA for a particular disorder could be considered safe without requiring full clinical trials to treat each mutant allele. For safer knock-ins, CRISPR-based transposon systems seem poised to overtake nucleases for this function (Klompe et al., 2022; Pallarès-Masmitjà et al., 2021). Nucleases might be considered a “first-generation” technology for targeted insertion of large sequences such as genes because they only create a double-strand break and leave subsequent steps to the many DNA repair pathways in the cell, which is the source of unexpected events. “Second-generation” CRISPR-guided transposon, recombinase and integrase systems perform both break and repair steps, and again benefit from improved delivery systems for their larger payloads. The challenge will be the efficiency and specificity of these systems compared to traditional nuclease-based homology directed repair. For epigenetic editors, the challenge lies in whether life-long changes in target gene expression will require continued expression of the epigenetic editor, or if a short-term treatment can cause a long-term change in the epigenetic information so that expression of the editor is no longer required. The latter might avoid concerns about immune responses to the foreign editor, which will be much more sensitive in humans than in mice, as well as allay concerns about longevity of expression. For RNA editors, the challenge is avoiding collateral damage. It was long known that Cas13 proteins *in vitro* and in bacteria possessed the unusual property of “collateral activity”; once the single-stranded RNA target was cleaved, the enzyme became a non-specific RNase. The collateral activity has been exploited as a system for the sensitive *in-vitro* detection of specific RNAs, such as the SARS-CoV-2 viral RNA by the SHERLOCK system (Gootenberg et al., 2017). Fortuitously, early studies reported the absence of collateral activity in mammalian cells (Abudayyeh et al., 2017; Konermann et al., 2018). Although the mechanism for the loss of this activity was never clear, Cas13 editing was shown to knock down expression of specific mRNAs in mouse models with no apparent adverse effects (Blanchard et al., 2021; Powell et al., 2022). However, more recent studies have found compelling evidence of significant collateral damage of other cellular RNAs in eukaryotic cells (Wang et al., 2019; Ai et al., 2022). This new evidence may lead to a shift away from Cas13 as an RNA nuclease to systems that lack inherent collateral activity, such as the Cas7-11 system (Özcan et al., 2021).

## The most threatening challenge

However, there is one challenge that poses an existential threat to the burgeoning field of therapeutic genome editing above all others, and that is cost. As a particularly cautionary example, Glybara, a gene therapy for lipoprotein lipase deficiency, was approved as the first gene therapy in Europe in 2012 but was removed after 2 years on the EU market in 2017 due to poor sales (only a single person was treated outside of a clinical trial). At a cost of ∼$1,000,000 per treatment, it was called the most expensive drug in history (technologyreview, 2022), and that was not a price that payers were willing to pay. As we consider the many technical challenges to create genome engineering therapies described above and elsewhere, the realization that the promise of therapy can fail for business reasons is sobering. Yet the first gene therapy approved in the USA, Luxturna, approved in 2017 to treat Leber’s congenital amaurosis, costs ∼$850,000 to treat both eyes (Darrow, 2019). Zolgensma, approved in the USA in 2019 to treat spinal muscular atrophy, costs ∼$2,125,000 per treatment (Garrison et al., 2021). Such prices give pause as to whether gene editing treatments for the 7,000 rare diseases will realistically be accessible to those who urgently need them, or if the limited demand would justify therapeutic development at all. Will the healthcare system overall be able to afford it? Some suggest that, just as the first cars and computers were expensive but are now readily affordable, prices will naturally come down as more gene and gene editing therapies become available. Unfortunately, there are reasons to doubt this scenario. The scenario assumes that prices are primarily based on “cost of goods sold”, the direct cost of producing the goods. However, most gene therapies are not valued based on cost of goods sold; rather, they are valued based on calculations such as quality-adjusted-life-years gained by treatment, and how much less expensive the treatment appears compared to life-long drug or molecular therapy (Zimmermann et al., 2019; Dean et al., 2021). For example, the high cost of ∼$15, 000, 000 for treating a hemophilia A patient with factor VIII over the course of their life has been used to justify the comparatively lower cost of Zolgensma (Garrison et al., 2021). Such valuations based on more expensive treatments or monetization of quality-adjusted-life-years are not likely to change due to a reduction in cost of goods. Thus, the promise of gene editing therapies seems so close from a technological perspective, yet so perilously far from a financial perspective.

In conclusion, perhaps for too long the starry-eyed aspirations of technologies developed by academic scientists have been separated from the real-world financial issues involved in translating discoveries into treatments. It is not too soon to ask the question as to whether the current system will serve us well, or if better paradigms could be envisioned. If we are to realize the promise of gene editing therapies, it will likely require academics and industry, foundations and patient advocates, and clinicians and payers to understand each other better. *Frontiers in Genome Editing* is dedicated to giving a forum to all of these stakeholders. We welcome your input and perspective in this important conversation.
